# Contact Dermatitis Due to Nickel Allergy in Patients Suffering from Non-Celiac Wheat Sensitivity

**DOI:** 10.3390/nu9020103

**Published:** 2017-02-02

**Authors:** Alberto D’Alcamo, Pasquale Mansueto, Maurizio Soresi, Rosario Iacobucci, Francesco La Blasca, Girolamo Geraci, Francesca Cavataio, Francesca Fayer, Andrea Arini, Laura Di Stefano, Giuseppe Iacono, Liana Bosco, Antonio Carroccio

**Affiliations:** 1 Dipartimento di Biologia e Medicina Interna e Specialistica (DiBiMIS), Internal Medicine Unit, University Hospital, Palermo 90100, Italy; adalcamo@hotmail.it (A.D.); pasquale.mansueto@unipa.it (P.M.); maurizio.soresi@unipa.it (M.S.); iacobuccirosario@gmail.com (R.I.); francescolablasca@gmail.com (F.L.B.); francesca.fayer@libero.it (F.F.); lauradist@virgilio.it (L.D.S.); 2 Surgery Department, University Hospital, Palermo 90100, Italy; girolamo.geraci@unipa.it; 3 Pediatric Unit, “Giovanni Paolo II” Hospital, Sciacca (ASP Agrigento) 90100, Italy; francesca_cv@inwind.it; 4 DiBiMIS, Gastroenterology Unit, University Hospital, Palermo 90100, Italy; a.arini@libero.it; 5 Pediatric Gastroenterology Unit, “ARNAS Di Cristina” Hospital, Palermo 90100, Italy; stoai@inwind.it; 6 Dipartimento di Scienze e Tecnologie Biologiche Chimiche e Farmaceutiche (Ste.Bi.CeF), University of Palermo, Palermo 90100, Italy; liana.bosco@unipa.it

**Keywords:** non-celiac wheat sensitivity, nickel allergy, cutaneous symptoms, irritable bowel syndrome

## Abstract

Background: Non-celiac wheat sensitivity (NCWS) is a new clinical entity in the world of gluten-related diseases. Nickel, the most frequent cause of contact allergy, can be found in wheat and results in systemic nickel allergy syndrome and mimics irritable bowel syndrome (IBS). Objective: To evaluate the frequency of contact dermatitis due to nickel allergy in NCWS patients diagnosed by a double-blind placebo-controlled (DBPC) challenge, and to identify the characteristics of NCWS patients with nickel allergy. Methods: We performed a prospective study of 60 patients (54 females, 6 males; mean age 34.1 ± 8.1 years) diagnosed with NCWS from December 2014 to November 2016; 80 age- and sex-matched subjects with functional gastrointestinal symptoms served as controls. Patients reporting contact dermatitis related to nickel-containing objects underwent nickel patch test (Clinicaltrials.gov registration number: NCT02750735). Results: Six out of sixty patients (10%) with NCWS suffered from contact dermatitis and nickel allergy and this frequency was statistically higher (*p* = 0.04) than observed in the control group (5%). The main clinical characteristic of NCWS patients with nickel allergy was a higher frequency of cutaneous symptoms after wheat ingestion compared to NCWS patients who did not suffer from nickel allergy (*p* < 0.0001). Conclusions: Contact dermatitis and nickel allergy are more frequent in NCWS patients than in subjects with functional gastrointestinal disorders; furthermore, these patients had a very high frequency of cutaneous manifestations after wheat ingestion. Nickel allergy should be evaluated in NCWS patients who have cutaneous manifestations after wheat ingestion.

## 1. Introduction

In recent years a new clinical entity has emerged which includes patients who consider themselves to be suffering from problems caused by wheat and/or gluten ingestion, even though they do not have celiac disease (CD) or wheat allergy. This clinical condition has been named non-celiac gluten sensitivity (NCGS) [1,2,3], although in a recent article we suggested the term “non-celiac wheat sensitivity” (NCWS) [4], because to date it is not known what component of wheat actually causes the symptoms. Other areas of doubt in NCWS regard its pathogenesis; while some papers have reported intestinal immunologic activation [5,6,7,8,9], others have linked NCWS to the dietary short–chain carbohydrate load (fermentable oligo-di-monosaccharides and polyols: FODMAPs) [10,11]. We recently demonstrated that a high percentage of patients with NCWS develop autoimmune disorders and are antinuclear antibody (ANA) positive, supporting an immunologic involvement in NCWS [12]. Furthermore, some papers have also reported a high frequency (22% to 35%) of coexistent atopic diseases in NCWS patients [13,14], and we suggested that a percentage of NCWS patients could actually suffer from non-immunoglobulin E (IgE)-mediated wheat allergy [15]. Nickel is the fourth most used metal and the most frequent cause of contact allergy in the industrialized world. As a natural element of the earth’s crust, small amounts are found in water, soil, and foods, including wheat. Nickel allergy not only affects the skin but can also result in systemic manifestations. Systemic nickel allergy syndrome (SNAS) can have signs and symptoms that are cutaneous (urticaria/angioedema, flares, itching), and/or gastrointestinal (meteorism, colic, diarrhea) [16]. It has been reported that 15% of NCWS patients suffered from allergy to nickel [13], but that study did not further characterize this subgroup of patients, and the NCWS diagnosis was not reached by means of a double-blind placebo-controlled (DBPC) challenge as recommended [3]. The present study was designed to: (A) evaluate the frequency of contact dermatitis due to nickel allergy in NCWS patients diagnosed by a DBPC challenge; and (B) identify the clinical, serological, and histological characteristics of NCWS patients who were positive for nickel allergy compared to NCWS patients without nickel allergy.

## 2. Materials and Methods

### 2.1. Study Design and Population

We prospectively included adult patients with functional gastroenterological symptoms according to the Rome III criteria [17], and a definitive diagnosis of NCWS. The patients were recruited between December 2014 and November 2016 at three centres: (1) Department of Internal Medicine at the University Hospital in Palermo; (2) Department of Internal Medicine of the Hospital of Sciacca; (3) the Gastroenterology Units of the “ARNAS Di Cristina” Hospital in Palermo. These patients were randomly selected, by a computer generated method, among those who were cured by elimination diet with the exclusion of wheat and tested positive to the DBPC diagnostic wheat challenge. Sixty NCWS patients (54 females, 6 males; mean age 34.1 ± 8.1 years) were included in the study. Fifty-five patients had reported gastrointestinal symptoms that could be related to wheat ingestion; five others had showed fibromyalgia-like symptoms and/or anaemia, without gastrointestinal symptoms. The characteristics of the NCWS patients suffering from nickel allergy were compared to those of the NCWS patients who did not suffer from nickel allergy. A control group of 80 patients with functional gastroenterological symptoms was selected to compare the frequency of nickel allergy in NCWS and non-NCWS patients. These controls were randomly chosen by a computer-generated method from subjects diagnosed during the same period, age-matched (±2 years) and sex-matched (±5%) to the NCWS patients. The controls had undergone the same elimination diet as the NCWS patients and had not shown any clinical improvement.

### 2.2. Diagnostic Criteria

NCWS diagnosis. All subjects met the recently proposed criteria [1]: negative serum anti-tissue transglutaminase and anti-endomysium (EmA) IgA and IgG antibodies; absence of intestinal villous atrophy; IgE-mediated immunoallergy tests negative for wheat (skin prick tests and/or serum–specific IgE detection). Additional criteria for our patients were: resolution of the gastrointestinal symptoms on a standard elimination diet, without wheat, cow’s milk, egg, tomato, chocolate, or other food(s) causing self-reported symptoms; as well as symptom reappearance on a DBPC wheat challenge, performed as described previously [9]. As in previous studies, a DBPC cow’s milk protein challenge and other “open” food challenges were also performed (see Supplementary Materials for details about the elimination diet and the DBPC challenge method). Exclusion criteria were: age <18 years; positive EmA in the culture medium of the duodenal biopsies, even if the villi–to–crypts ratio in the duodenal mucosa was normal; self-exclusion of wheat from the diet and refusal to reintroduce it before entering the study; other organic cutaneous and/or gastrointestinal diseases; and concomitant treatment with steroids and/or antihistamines.

Contact dermatitis diagnosis. Allergic contact dermatitis was diagnosed by an independent physician who blindly reviewed the skin in all the subjects included in the study. The diagnosis was posed in patients with local eczematous lesions on the skin in close contact with nickel-containing objects. Suspected systemic nickel allergy syndrome (SNAS) was defined as a reaction characterized not only by diffused eczematous lesions (systemic contact dermatitis), but also by extracutaneous signs and symptoms, mainly gastrointestinal, after ingestion of nickel–rich foods (i.e., tomato, cocoa, beans, mushrooms, vegetables, wheat flour, etc.) [16]. In all cases, the diagnosis was confirmed by means of the epicutaneous patch test which provoked delayed lesions.

Associated autoimmune disease diagnosis. A structured and previously validated questionnaire [12] was used to diagnose autoimmune diseases.

Atopic disease diagnosis. As in previous studies, the following diseases were diagnosed according to standard criteria: rhinitis, conjunctivitis, bronchial asthma, and atopic dermatitis.

### 2.3. Laboratory Methods

Serology for CD, duodenal histology studies, and Human Leucocyte Antigens (HLA)-DQ typing were performed on all patients to exclude a CD diagnosis, as described previously (Supplementary Materials, [13,14]).

Nickel allergy. All of the NCWS patients reporting contact dermatitis related to nickel-containing objects were patch tested. Patch tests were performed in our laboratory, with the Italian Society of Allergological, Occupational and Environmental Dermatology (SIDAPA) standard series (Lofarma S.p.A., Milano, Italy), using a commercial method (Curatest^®^ F, Lohmann & Rauscher; Neuwied, Germany); allergens were applied on the upper back and removed after 72 h. The sites were examined on removal and 24 h or 48 h after removal according to the recommended International Contact Dermatitis Research Group guidelines. Reactions were graded as: negative; macular erythema; weak reaction (non-vesicular erythema, infiltration or papules); strong reaction (edema or vesicles); extreme reaction (spreading, bullous and ulcerative lesions); or irritant. The treating physician determined the relevance of each positive result on the basis of the patient’s history and known exposure. Weak, strong, and extreme reactions at the final reading were considered positive reactions.

Duodenal histology. Duodenal histology was classified according to Corazza and Villanacci [18].

Serum anti-nuclear antibodies (ANA). ANA were identified by Human epithelial type 2 (HEp-2) cells, using an indirect immunofluorescence technique; a titer of 1:40 or higher was considered positive and the sera were titered at progressive dilutions until they became negative.

### 2.4. Statistical Analysis

Data were expressed as mean ± Standard deviation (SD) when the distribution was Gaussian and differences were calculated using the Student *t* test. Otherwise, data were expressed as median and range and analyzed using the Mann-Whitney U test. Fisher’s exact or the χ^2^ tests were used where appropriate. *p* < 0.05 was considered significant. All analyses were performed using the SPSS software package (version 16.0, released 2007, SPSS Inc., Chicago, IL, USA). The study protocol conformed to the ethical guidelines of the Declaration of Helsinki, and was approved by our institution’s human research committee (University Hospital of Palermo, identification code 4/2015), and registered at clinicaltrials.gov (registration number: NCT02750735).

## 3. Results

Table 1 shows the clinical characteristics of the patients, compared to the control group composed of patients with functional gastroenterological disorders who did not improve on the elimination wheat-free diet. In general, in NCWS patients there was a significantly higher percentage of self-reported wheat intolerance and coexisting atopic diseases than in IBS controls.

### 3.1. Frequency of Contact Dermatitis Due to Nickel Allergy

Six (10%) of the 60 NCWS patients reported contact dermatitis related to nickel-containing objects and all tested positive on nickel patch. In the control group contact dermatitis related to nickel-containing objects was observed in 4 out of 80 patients (5%), and nickel patch was positive in all. This different frequency was statistically significant (*p* = 0.04).

### 3.2. Clinical Characteristics of the NCWS Patients with Nickel Allergy

Table 2 summarizes the clinical characteristics of the NCWS patients with associated contact dermatitis and nickel allergy compared to NCWS patients who did not suffer from contact dermatitis and nickel allergy. Patients with NCWS and nickel allergy had a higher frequency of cutaneous symptoms, considered as a whole, after wheat ingestion, than NCWS patients who did not have associated nickel allergy (100% vs. 7%; *p* < 0.0001). In particular, cutaneous erythema after wheat ingestion was present in all NCWS patients suffering from nickel allergy, whereas widespread itching and urticaria were observed in 50% and 33% of them respectively. In the NCWS patients without nickel allergy, all of the above cutaneous symptoms were present in less than 10% of the cases. No other clinical characteristics were different in the two groups, including the frequency and kind of gastrointestinal symptoms, the associated atopic and autoimmune diseases, and multiple food hypersensitivity, which showed a trend to a higher frequency in NCWS patients with nickel allergy (83% vs. 46% in NCWS without nickel allergy; *p* = 0.07).

## 4. Discussion

NCWS is a relatively new clinical entity in the world of “gluten-related disease” [1,19,20], although its pathogenesis remains uncertain [20,21]. A role for FODMAP malabsorption as a determinant of the abdominal symptoms in NCWS patients has been advocated [10,11], and wheat is actually one of the foods richest in FODMAPs, but FODMAP intolerance cannot explain both the extra-intestinal symptoms and the increasing evidence of immunologic involvement in many NCWS patients [5,7,8,9,22,23].

The present study focused attention on the frequency of contact dermatitis and nickel allergy in NCWS and the clinical characteristics of the subjects who had this association. We found a 10% frequency of contact dermatitis and nickel allergy in NCWS, which is statistically higher than observed in the control group composed of IBS patients who did not suffer from NCWS (whose symptoms were not improved on the elimination diet, with the exclusion of wheat). This finding is consistent with our previous observation that one third of NCWS patients with an IBS-like clinical manifestation had associated atopic diseases [14], and the high prevalence of atopic diseases (42%) observed in the patients involved in the present study. Furthermore, an Italian multicentre study of about 500 patients found that more than 20% of suspected NCWS patients had an allergy to one or more inhalants (26% to mites), food, or metals [13].

Regarding the clinical characteristics of the patients with NCWS associated with nickel allergy, we found that they had a significantly higher frequency of cutaneous manifestations after wheat ingestion than NCWS patients who did not suffer from contact dermatitis and nickel allergy. In particular, cutaneous erythema was present in all of the patients. In contrast, cutaneous manifestations were present in only 7% of the NCWS patients not suffering from nickel allergy. On this basis it could be suggested that the patients with NCWS who have cutaneous symptoms after wheat ingestion should be investigated for suspected nickel allergy.

Some limits of our study must be mentioned. Our study design did not permit evaluation of the frequency of nickel allergy in NCWS as we performed the nickel patch only with the patients who reported contact dermatitis. Other NCWS patients could have suffered from nickel allergy without contact dermatitis signs. We studied patients who were referred to tertiary centres with experience in CD and NCWS, so this created a selection bias. Consequently, our results cannot be extended to the broad population of self-treated or diagnosed NCWS patients. The sample size of NCWS patients was relatively small and it must be remembered that the general prevalence of nickel allergy in western countries is high and similar to the prevalence reported in NCWS patients in our study [24].

Furthermore, we have not performed any study to evaluate the hypothesis that nickel allergy could contribute to the pathogenesis of NCWS. It has been demonstrated that some immunologic pathways involved in the nickel-induced mucositis and dermatitis, i.e., the inflammatory response via the activation of TLR4 and the infiltration of lymphocytes that secrete Interleukin (IL)-17 and Interferon-gamma (IFN)-γ [25,26], have also been supposed or demonstrated in NCWS [5,7,8,9,27].

The strength of our data are the patient selection based on a NCWS diagnosis made by using the DBPC challenge method, and the study design specifically constructed to reveal the presence of nickel allergy.

## 5. Conclusions

In conclusion, our study suggests that contact dermatitis due to nickel allergy is more frequent in NCWS patients than in subjects with functional gastrointestinal disorders. Furthermore, as these patients had a very high frequency of cutaneous manifestations after wheat ingestion, we suggest that NCWS patients who have cutaneous symptoms should be investigated for suspected nickel allergy.

## Figures and Tables

**Table 1:** Clinical characteristics of the NCWS patients compared to the control group composed of patients suffering from IBS who did not improve on elimination diet.

	NCWS Patients (*n* = 60)	IBS Controls (*n* = 80)	*p* Value
Sex	54 F, 6 M	72 F, 8 M	Not Applicable, matching factor
Age	34.1 ± 8.1 years	35.4 ± 9.0 years	Not Applicable, matching factor
Self-reported wheat intolerance	33 cases (55%)	20 cases (25%)	0.05
Family history of celiac disease	6 cases (10%)	2 cases (2.5%)	0.07
Coexistent atopic diseases	25 cases (42%)	7 cases (8.7%)	0.0001

Note: Family history of CD indicates a CD diagnosis in a first-degree relative. NCWS, non-celiac wheat sensitivity; IBS, irritable bowel syndrome; F, Female; M, Male; CD, celiac disease.

**Table 2:** Clinical characteristics of the NCWS patients with contact dermatitis due to nickel allergy (*n* = 6) compared to NCWS patients who did not suffer from contact dermatitis (*n* = 54).

	NCWS without Contact Dermatitis *n* = 54 (90%)	NCWS with Contact Dermatitis and Nickel Allergy *n* = 6 (10%)	*p* Value
Male Sex	5 (9.3%)	1 (16.6%)	not significant
Age (years) (x ± SD)	35.0 ± 8.1	33.8 ± 9.2	not significant
Symptom Duration (months; median and range)	70 (6–240)	66 (3–216)	not significant
Coexistent Atopic Diseases	20 (37%)	5 (83%)	0.07 ns
Coexistent Autoimmune Diseases	15 (28%)	2 (33%)	not significant
Abdominal Bloating	48 (89%)	6 (100%)	not significant
Abdominal Pain	45 (83%)	6 (100%)	not significant
Diarrhea	32 (59%)	4 (66%)	not significant
Constipation	11 (20%)	1 (17%)	not significant
Vomit	5 (9%)	1 (17%)	not significant
GERD-Like Symptoms	26 (48%)	3 (50%)	not significant
Extra-intestinal Symptoms	37 (68%)	5 (83%)	not significant
Cutaneous Symptoms after Wheat Ingestion	4 (7%)	6 (100%)	0.0001
Diffuse Itching	3 (5%)	3 (50%)	0.002
Cutaneous Erythema	4 (7%)	6 (100%)	0.0001
Urticaria	3 (5%)	2 (33%)	not significant
Multiple Food Hypersensitivity	25 (46%)	5 (83%)	not significant
Serum ANA Positivity	18 (33%)	2 (33%)	not significant
HLA DQ2 or DQ8 Positive	27 (50%)	3 (50%)	not significant
Increased Number of IEL in Duodenal Mucosa (Grade A Histology)	29 (54%)	3 (50%)	not significant

SD: standard deviation; ns: not significant; GERD: gastro-esophageal reflux disease; HLA: Human Leucocyte Antigens; IEL: intra-epithelial lymphocytes; ANA, anti-nuclear antibodies.

## Supplementary Materials

The following are available online at http://www.mdpi.com/2072-6643/9/2/103/s1.

## Abbreviations

ANA: anti-nuclear antibodies; anti-tTG: anti-tissue transglutaminase; CD: celiac disease; DBPC: double-blind placebo-controlled; EmA: anti-endomysium antibodies; FODMAPs: fermentable oligo-di-monosaccharides and polyols; IBS: irritable bowel syndrome; NCGS: non-celiac gluten sensitivity; NCWS: non-celiac wheat sensitivity; SD: standard deviation; SNAS: systemic nickel allergy syndrome.
