# Supplementary material for: Contact Dermatitis Due to Nickel Allergy in Patients Suffering from Non-Celiac Wheat Sensitivity
Source: Nutrients. 2017 Feb 2;9(2):103. doi: 10.3390/nu9020103 (PMC5331534; doi:10.3390/nu9020103)
Supplement: Supplementary file 1 [file nutrients-09-00103-s001.docx]

Supplementary Materials: Contact Dermatitis due to Nickel Allergy in Patients Suffering from Non-Celiac Wheat Sensitivity

Alberto D’Alcamo, Pasquale Mansueto, Maurizio Soresi, Rosario Iacobucci, Francesco La Blasca, Girolamo Geraci, Francesca Cavataio, Francesca Fayer, Andrea Arini, Laura Di Stefano,
Giuseppe Iacono, Liana Bosco and Antonio Carroccio

Elimination Diet and Double-Blind Placebo-Controlled (DBPC) Challenge

On entering the study all patients commenced an identical standard elimination diet, with the exclusion of wheat, cow’s milk, eggs, tomato and chocolate. Patients self-reporting food hypersensitivity were also asked to avoid ingestion and/or contact with the food(s) causing symptoms. Food diaries were maintained by the patients during the elimination diet period to assess dietary intake and adherence to the diet. After four weeks on elimination diet the patients who reported symptom resolution underwent DBPC challenges.

The challenges were performed with the reintroduction of a single food at a time. Patients were randomized to receive either the “active food” or the placebo, according to a computer-generated order, determined by an observer not involved in the study. In the case of wheat, the DBPC challenge was performed with packets of flour coded A or B containing wheat flour or rice flour, respectively. Packets A or B were given for 2 consecutive weeks and then after 1 week of washout the patients received the other packets for another 2 weeks (cross-over design). Wheat challenge was performed administering a daily dose of 80 g of flour, which was dissolved and cooked by the patients themselves. Wheat packets contained 6.5 g of gluten. DBPC for cow’s milk was performed by administering capsules coded as A or B containing milk proteins (casein from bovine milk, lactoalbumin, lactoglobulin—daily dose 6 g, equal to about 200 mL of cow’s milk) or xylose, respectively. A total of six capsules daily were given three times daily, away from meals. During all phases of the study, including the challenge period, the severity of symptoms was recorded: the patients completed a 100 mm visual analog scale with 0 representing no symptoms, which assessed overall symptoms and the specific symptoms they each reported. The challenges were stopped when clinical reactions occurred (increase in visual anlogue scale (VAS) score >30) for at least two consecutive days (onset of abdominal discomfort or pain, associated with a change in stool frequency and/or stool appearance). The challenges were considered positive if the same symptoms which had been initially present reappeared after their disappearance on the elimination diet. Patients with a similar clinical reaction during the “placebo period” were not considered affected by Non-celiac wheat sensitivity (NCWS), irrespective of their worsening during the period in which they consumed capsules containing wheat. The codes of the packets and capsules were broken only at the end of the study so the investigators did not know their content during the study period. Challenges for other foods in patients with suspected multiple food hypersensitivity were performed in an open fashion.

Laboratory Methods and Criteria for Celiac Disease (CD) Diagnosis

On entering the study all patients underwent serum anti-tissue transglutaminase (anti-tTG) immunoglobulin A (IgA) and IgG, anti-endomysium antibodies (EmA) IgA, and anti-gliadin (AGA) IgA and IgG assays, performed using commercial kits (Eu-tTG IgA, anti-endomysium, and anti-gliadin IgA and IgG, Eurospital Pharma; Trieste, Italy). Patients were also typed for Human Leucocyte Antigens (HLA)-DQ phenotypes by polymerase chain reaction using sequence-specific primers, with a rapid method (DQ-CD Typing Plus by BioDiaGene, Palermo, Italy). CD diagnosis was excluded in patients with negative serum antibody assays and not carrying the DQ2/DQ8 haplotypes.

Those patients who reported symptom disappearance on a wheat-free diet and carried the DQ2 and/or the DQ8 haplotypes underwent duodenal biopsies irrespective of the serum CD–specific antibody results. The biopsies were performed when the patients were on a regular wheat-containing diet. CD diagnosis was excluded in these patients only in the absence of villi atrophy and anti-endomysium antibody production in the culture medium of the duodenal mucosa (Anti-endomysium biopsy, Eurospital Pharma; Trieste, Italy).
